# Role of large language models in mental health research: an international survey of researchers’ practices and perspectives

**DOI:** 10.1136/bmjment-2025-301787

**Published:** 2025-06-12

**Authors:** Jake Linardon, Mariel Messer, Cleo Anderson, Claudia Liu, Zoe McClure, Hannah K. Jarman, Simon B. Goldberg, John Torous

**Affiliations:** 1 SEED Lifespan Strategic Research Centre, School of Psychology, Faculty of Health, Deakin University, Geelong, Victoria, Australia; 2 Department of Counselling Psychology, University of Wisconsin, Madison, Wisconsin, USA; 3 Center for Healthy Minds, University of Wisconsin, Madison, Wisconsin, USA; 4 Division of Digital Psychiatry, Department of Psychiatry, Beth Israel Deaconess Medical Center, Harvard Medical School, Boston, Massachusetts, USA

**Keywords:** cross-sectional studies, psychiatry, machine learning

## Abstract

**Background:**

Large language models (LLMs) offer significant potential to streamline research workflows and enhance productivity. However, limited data exist on the extent of their adoption within the mental health research community.

**Objective:**

We examined how LLMs are being used in mental health research, the types of tasks they support, barriers to their adoption and broader attitudes towards their integration.

**Methods:**

714 mental health researchers from 42 countries and various career stages (from PhD student, to early career researcher, to Professor) completed a survey assessing LLM-related practices and perspectives.

**Findings:**

496 (69.5%) reported using LLMs to assist with research, with 94% indicating use of ChatGPT. The most common applications were for proofreading written work (69%) and refining or generating code (49%). LLM use was more prevalent among early career researchers. Common challenges reported by users included inaccurate responses (78%), ethical concerns (48%) and biased outputs (27%). However, many users indicated that LLMs improved efficiency (73%) and output quality (44%). Reasons for non-use were concerns with ethical issues (53%) and accuracy of outputs (50%). Most agreed that they wanted more training on responsible use (77%), that researchers should be required to disclose use of LLMs in manuscripts (79%) and that they were concerned about LLMs affecting how their work is evaluated (60%).

**Conclusion:**

While LLM use is widespread in mental health research, key barriers and implementation challenges remain.

**Clinical implications:**

LLMs may streamline mental health research processes, but clear guidelines are needed to support their ethical and transparent use across the research lifecycle.

**WHAT IS ALREADY KNOWN ON THIS TOPIC**

- Large language models (LLMs) have shown value in supporting academic tasks such as writing and coding, with widespread uptake in fields like education and medicine; however, their use in mental health research remains underexplored, and concerns about accuracy, bias and data privacy continue to limit adoption.

**WHAT THIS STUDY ADDS**

- This is the first large-scale survey to examine how mental health researchers are using LLMs.
- Findings reveal high uptake, particularly among early career researchers, with most using LLMs for proofreading and coding.
- Ethical concerns, accuracy issues and lack of training were the most reported barriers.

**HOW THIS STUDY MIGHT AFFECT RESEARCH, PRACTICE OR POLICY**

- The results highlight the need for clearer institutional guidelines, training and disclosure practices to support responsible LLM use in research.
- Journals and institutions may use these insights to develop policies that balance innovation with integrity in mental health science.

## Background

 Integrating artificial intelligence (AI) into sectors such as health, engineering and marketing has brought transformative changes, with large language models (LLMs) at the forefront due to their impressive capabilities and growing accessibility. While AI encompasses a broad range of technologies, such as machine learning algorithms for predictive modelling, computer vision for medical image analysis, and robotics for automating surgical procedures,^1^ LLMs represent a specific subset of AI tools designed to understand and generate text. These models often perform tasks such as answering questions, summarising content and translating languages with a level of fluency that closely resembles human communication.^2^ Despite promising applications of LLMs that range from automating content creation to enhancing decision-making and streamlining communication, concerns remain about data privacy, misinformation and output transparency.^3^ LLMs have been widely studied in education,^4^ medicine^5^ and engineering research,^6^ yet their empirical investigation within mental health settings has received comparatively less attention.

LLMs could enhance the delivery of mental healthcare.^7^ By analysing vast amounts of data derived from clinical case notes, session transcripts, social media communication and mobile device interactions, LLMs could assist with the diagnosis, monitoring, prevention and treatment of mental disorders.^8^ They are also capable of streamlining administrative tasks (eg, booking appointments, note writing, billing, etc) for clinicians, allowing more time and resources to be devoted to patient care.^9^ Despite the nascency of this field, emerging pilot data provide promising evidence supporting the potential of LLMs to perform these functions effectively.^10^,^12^

While empirical investigations of LLMs in mental health have largely concentrated on clinical settings,^8 13^ their potential to support academic research remains underexplored despite being potentially more fruitful.^14^ With their capabilities in language generation, information synthesis and task automation, LLMs may be well-suited to assist with various research-related tasks, such as proofreading manuscripts, refining code, supporting peer review, conducting literature reviews, interpreting data analyses and generating research questions and hypotheses. These capabilities may appeal to researchers operating under increasing pressure to publish high volumes of work within short timeframes to remain competitive for academic promotions, funding opportunities and research recognition.^15^

Given the potential of LLMs to streamline a wide range of research-related tasks, enhance productivity and potentially create new risks, it is important to understand how—and to what extent—mental health researchers are engaging with these tools. Investigating current practices and perspectives of LLMs can provide crucial insights into whether these tools are being used to support academic research, by whom and for what purposes. Equally important is identifying barriers to their adoption, challenges encountered in their use, and broader attitudes towards their integration in this specialised field. This is particularly timely, as LLM capabilities have advanced considerably in recent months with the introduction of features such as multimodal inputs, improved contextual understanding and enhanced tools for citation, summarisation, and coding support.^14^ Insights into LLM use are necessary to ensure ethical and effective integration into research workflows, guide the development of institutional guidelines for responsible use and promote high standards of transparency, safety and performance. These issues are especially salient for mental health researchers, who often work with highly sensitive personal health data and must communicate complex psychological constructs that may be less common in other scientific fields. Thus, a focused investigation into the current practices, purposes of use, challenges and broader attitudes surrounding LLM adoption among mental health researchers is necessary.

### Objectives

To address this gap, the present study examined current practices and perspectives surrounding LLM use among mental health researchers. Specifically, we sought to understand the extent to which LLMs are being used to support mental health research, the types of tools and tasks they perform, the challenges encountered in their use, barriers to adoption, and broader attitudes towards their integration within this specialised field.

## Methods

### Design

A cross-sectional online survey, adhering to the Strengthening the Reporting of Observational Studies in Epidemiology guidelines (see online supplemental material), was delivered via Qualtrics to researchers whose self-identified primary focus was on mental health research. Recruitment occurred between 18 March 2025 and 5 April 2025.

### Participants and procedure

Eligible participants were active researchers (including PhD students) whose self-identified primary focus was on mental health sciences. We searched through several journals listed in Scimago with the subject categories of “Psychiatry and Mental Health” and “Clinical Psychology”, and generated a database of corresponding author emails from publications featured in these journals over the past 5 years. After removing duplicate and inactive emails, we sent approximately 4000 invitations to corresponding authors from this list. The email invitation indicated that the survey assessed researchers’ use of and opinions surrounding LLMs to assist with the conduct of mental health research, rather than general AI systems (eg, statistical modelling or image analysis). We specified that no prior experience in or knowledge of AI was required. We received 749 responses to the invitation, reflecting a 19% response rate. Thirty-five participants were screened out of the study because they reported that their research focus was not primarily on mental health. Thus, 714 researchers met full inclusion criteria and were included in the analyses. The online survey took between 5 and 10 min to complete. No compensation was offered.

### Measures

A web-based survey was designed to explore mental health researchers’ use of and perspectives on LLMs in academic research. Items were developed for this study, informed by previous surveys of LLM use in other disciplines,^16 17^ key literature on LLM applications in mental health research^14^ and the author team’s expertise. The full survey is presented in the online supplemental figure 1.

#### Background characteristics

Participants provided demographic (eg, age, gender, country), academic (eg, qualification, affiliation, job title) and research background information, including years of experience, h-index, publication count, methodological expertise and primary research focus. They also reported their experience with AI in research.

#### LLM use and perspectives

Participants were first presented with the following lay definition of LLMs:

The following questions ask about your experience with and perspectives towards large language models (LLMs). LLMs are advanced artificial intelligence systems that can understand and generate human-like text. They are trained on vast amounts of written information and can respond to questions, summarize content, assist with writing, and generate ideas in a conversational way. Examples include ChatGPT, Google Gemini, and Claude.

Participants were then asked to indicate whether they had ever used an LLM to assist with any aspect of their academic research. Those who responded ‘no’ were asked to select from nine possible reasons for their non-use, which reflect commonly cited barriers to adopting LLMs in scientific research, such as lack of awareness, ethical concerns, limited technical skills and institutional restrictions. A free-text option was available for participants to specify other reasons not captured by the listed options. These participants were subsequently asked whether they would be more likely to use LLMs in their research (ie, ‘yes’, ‘no’ or ‘unsure’), if specific issues were addressed. Examples of the eight issues provided were clearer ethical guidelines for LLM use, improved accuracy and reliability and stronger data privacy and security assurances.

In contrast, participants who indicated that they had used LLMs for their research were asked to specify which LLMs they had used and how frequently they used LLMs for research tasks (ie, ‘daily’, ‘weekly’, ‘monthly’, ‘less than once a month’ or ‘only used once or twice’). They were then provided with a series of research tasks and were asked to select which of the following they had used LLMs to assist with. These tasks were divided into *writing and drafting tasks* (eg, proofreading and improving writing clarity, generating ideas for research questions or hypotheses, etc), *LLM-assisted code and scripting tasks* (eg, generating or refining code via natural language prompts, creating data visualisations, etc), *research process and methodology tasks* (eg, suggesting relevant measures, interpreting statistical findings, etc) and *administrative and logistical support tasks* (eg, summarising research meeting notes, assisting with science communication on social media, etc). A free-text option was available for participants to specify other purposes not captured by the listed options. LLM adopters were also provided with common challenges or limitations associated with their use (eg, inaccurate or misleading responses, lack of transparency about sources, over-reliance on AI-generated content, etc) and were asked to select which ones they had encountered (along with a free-text option). Finally, using a ‘yes’, ‘no’ or ‘unsure’ response option, LLM adopters were asked whether (1) LLMs had made their research process more efficient, (2) LLM use improved the quality of their research outputs, (3) they would recommend LLMs to colleagues for research support and (4) they would feel comfortable disclosing LLM use in academic work.

All participants were then asked to indicate their level of agreement using a 5-point scale (1=strongly disagree to 5=strongly agree) on seven items assessing their broader perspectives of LLM integration in mental health research. These items assessed attitudes pertaining to the anticipated role of LLMs in research, ethical and professional concerns and the need for institutional support and transparency.

### Data analysis

Descriptive statistics and percentages were presented for LLM adoption and broader attitudes. A multivariable logistic regression was also performed to explore factors associated with LLM adoption. In this model, age, gender, research productivity metrics (ie, h-index, years’ experience, publication count) and prior AI experience were entered as predictors simultaneously in the model. Predictors were considered significant at p<0.05.

## Findings

### Participant characteristics

A total of 714 mental health researchers completed the survey and were included in the analyses. Online supplemental table S1 provides a full breakdown of participant characteristics. The mean age of participants was 40.2 years (SD=11.48). The majority identified as women (59.1%), held a PhD or doctoral degree (76%) and reported psychology as their primary field of expertise (70.4%). Most were affiliated with a university (86.1%) and primarily engaged in quantitative research (84.3%). The largest proportions of participants resided in Europe (36%), followed by North America (32%), then Australia/Oceania (25%), Asia (5%), Africa and South America (<1%). In terms of research productivity, just over half reported an h-index between 0 and 30 and had (co)authored up to 60 publications. More than two-thirds had between 1 and 15 years of research experience. Participants most researched anxiety and related disorders (49.5%), depression (45.5%) and eating disorders (23.0%). Their primary research foci included treatment/intervention (60.6%), mechanisms of mental illness (48.6%), assessment/diagnosis (33.8%) and prevention/risk reduction research (31.6%). Regarding experience with AI, 18.5% reported none, 46.1% reported limited experience, 30.3% reported some experience and 5.2% reported extensive experience. Online supplemental figures S2 and S3 provide a more detailed overview of the sample characteristics.

### LLM use

When asked whether they had ever used LLMs to assist with their research, 496 (69.5%) participants responded ‘yes’ and 218 (30.5%) responded ‘no’.

Multivariable logistic regression was performed to examine factors independently associated with LLM use. The overall model was statistically significant (χ²=167.82 (df=6); p<0.001; Negelkerke R^2^=0.29). Fewer years of research experience (OR 0.67, 95% CI 0.53, 0.84) and more experience with AI (OR 3.73, 95% CI 2.84, 4.89) were significantly and uniquely associated with greater LLM adoption. Participant age (OR 1.00, 95% CI 0.98, 1.03), gender (OR 1.11, 95% CI 0.75, 1.65), h-index (OR 0.97, 95% CI 0.93, 1.01) and publication count (OR 0.95, 95% CI 0.84, 1.08) were not significantly associated with LLM adoption.

### Non-user perspectives

#### Reasons for non-use

**Figure 1:**
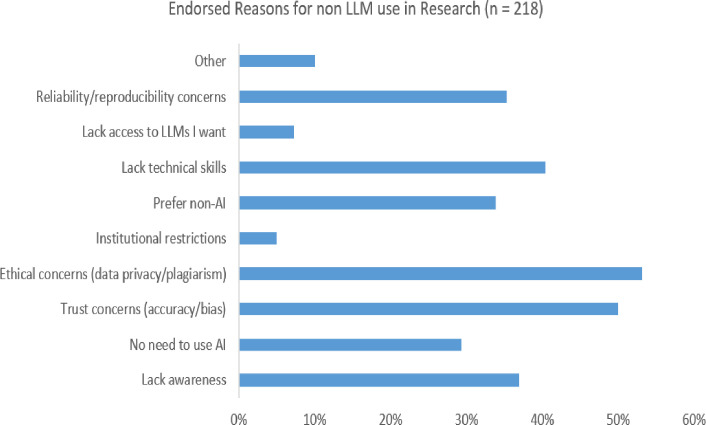
Reasons for researchers not using LLMs (n=218). AI, artificial intelligence; LLM, large language models.

Figure 1 shows the percentage of participants endorsing each reason for not using LLMs. The most endorsed reasons for non-use were ethical concerns about data privacy and plagiarism (n=116; 53.2%), concerns about accuracy and biases in LLM content (n=109; 50.0%), lack of technical skills (n=88; 40.4%) and lack of awareness of LLMs (n=81; 37.2%).

#### Conditions for increased LLM adoption among non-users

Online supplemental figure S4 presents the percentage of participants who indicated they would be more likely to use LLMs for research if certain issues were met. The most endorsed issues were increased transparency (n=138; 63.3%), more training or resources on LLM use (n=135; 61.9%), improved accuracy and reliability (n=129; 59.2%), clearer ethical guidelines (n=114; 52.3%) and better integration of LLMs into existing research tools and workflows (n=114; 52.3%).

### User perspectives

#### Types of models used

Of the 496 users, the most common models used were Open AI’s ChatGPT (n=468; 94.4%), followed by Microsoft’s Co-Pilot (n=86; 17.3%), Perplexity AI (n=54; 10.9%), Google’s Gemini (n=67; 13.5%) and Anthropic’s Claude (n=56; 11.3%). Other less commonly used models included Meta AI (n=19; 3.8%), X’s Grok (n=9; 1.3%), Deepseek (n=14; 2.8%), Undermind (n=3; <1%), Elicit (n=3; <1%) and GitHub Co-pilot (n=7; 1.4%).

#### Frequency of LLM use

In terms of frequency of use, most reported using LLMs ‘weekly’ (n=212; 42.7%), then ‘daily’ (n=101; 20.3%), ‘less than once a month’ (n=81; 16.3%), ‘monthly’ (n=53; 10.6%) and were ‘only used once or twice’ (n=49; 9.8%).

#### Purposes for using LLMs

Table 1 presents data on participants’ use of LLMs in their research. For *writing and drafting tasks*, the most frequently cited purposes for LLM use were proofreading or improving writing clarity (n=342; 69.0%) and summarising, synthesising or organising scientific literature (n=190; 38.3%). For *data cleaning and analysis*, the most common use was generating or refining code for analyses (n=246; 49.0%). Fewer than one-quarter of participants used LLMs for tasks related to *research methodology* or *administration and logistical support*.

**Table 1:** Purpose of LLM use in research among adopters (n=496)

Purpose	N endorsed (%)
Writing and drafting tasks	
Generating ideas for research questions or hypotheses	106 (21.4%)
Drafting or structuring research papers, abstracts or grants	173 (34.9%)
Proofreading or improving writing clarity	342 (69.0%)
Assisting with peer-review of papers or grant applications	56 (11.3%)
Creating education materials or presentations	140 (28.2%)
Summarising, synthesising or organising scientific literature	190 (38.3%)
LLM-assisted code and scripting tasks	
Assisting with data cleaning or preparation	69 (13.9%)
Generating or refining code for statistical analysis (R, Python, MATLAB)	246 (49.6%)
Analysing qualitative data (thematic analysis, summarising interview transcripts)	49 (9.9%)
Creating or refining data visualisations	62 (12.5%)
Research process and methodology	
Assisting with study design or methodology development	76 (15.3%)
Suggesting relevant measures, scales or assessments	76 (15.3%)
Interpreting or contextualising statistical findings or research outcomes	88 (17.7%)
Interpreting other academic research papers	87 (17.5%)
Administrative and logistical support	
Automating research-related tasks (formatting references, preparing tables)	93 (18.8%)
Supporting participant recruitment (drafting recruitment messages, generating survey items)	63 (12.7%)
Summarising meeting notes or research discussions	103 (20.8%)
Assisting with social media/science communication (drafting posts, lay summaries)	109 (22.0%)
Other (unique purposes not covered above referenced two times or more)	33 (6.7%)
Translation of research	4 (0.1%)
Drafting/Writing emails	4 (0.1%)
Generating title names/cover letters/journals to target	5 (0.1%)
Studying AI in mental health research	9 (1.8%)

AI, artificial intelligence; LLM, large language model.

#### Challenges encountered with LLM use

Table 2 presents the frequency of challenges participants encountered when using LLMs. The most reported issues were inaccurate or misleading responses (n=390; 78.6%), ethical concerns (n=227; 45.8%), biased outputs (n=134; 27.0%) and technical limitations (n=131; 26.4%). A small number of participants (n=18; 3.6%) reported no challenges encountered.

**Table 2:** Challenges encountered with LLM use in research among adopters (n=496)

Challenge	N endorsed (%)
Inaccurate or misleading responses	390 (78.6%)
Lack of transparency about sources and citations	298 (6.1%)
Ethical concerns (eg, data privacy/confidentiality, authorship, plagiarism)	227 (45.8%)
Bias in responses	134 (27.0%)
Over-reliance on AI-generated content	96 (19.4%)
Institutional or journal policies restricting LLM use	80 (16.1%)
Technical limitations (eg, token/context length, inability to handle large datasets)	131 (26.4%)
No challenges encountered	18 (3.6%)
Other (unique challenges not covered above referenced two times or more)	26 (5.2%)
Environmental impact	3 (<1%)
Financial limitations	2 (<1%)
Responses not useful	10 (2.0%)

AI, artificial intelligence; LLM, large language model.

#### Reflections on LLM impact and disclosure

Among users, when asked whether LLMs had made the research process more efficient, 360 (72.6%) responded yes, 102 (20.6%) were unsure and 34 (6.9%) responded no. When asked whether LLMs have improved the quality of their research outputs, 218 (44.0%) responded yes, 165 (33.3%) were unsure and 113 (22.8%) responded no. When asked if they would recommend LLMs to colleagues for research support, 369 (74.4%) responded yes, 105 (21.2%) were unsure and 22 (4.4%) responded no. When asked if they felt comfortable disclosing LLM use in academic work, 253 (51.0%) responded yes, 160 (32.3%) were unsure and 83 (16.7%) responded no.

### Broader perspectives of LLM integration in mental health research

Table 3 presents agreement ratings on broader perspectives of LLM integration in mental health research among the total sample. More than three-quarters agreed or strongly agreed that (1) LLMs will become a standard tool in research within the next 5–10 years (85.7%), (2) they would like more support or training on responsible LLM use (76.7%), (3) they have ethical concerns about LLM use in research in general (78.3%) and (4) researchers should be required to disclose LLM use in academic writing (79.8%). Just over half of the sample (60.4%) agreed or strongly agreed about having concerns that LLMs use could affect how their work was evaluated. However, less than half (43.0%) agreed or strongly agreed that LLMs will reduce the need for certain types of research roles.

**Table 3:** Attitudes towards LLM integration in mental health research among the total sample (n=714)

Item	Strongly disagree	Somewhat disagree	Neither agree/disagree	Somewhat agree	Strongly agree
LLMs will become a standard tool in academic research within the next 5–10 years	14 (2.0%)	32 (4.5%)	56 (7.8%)	294 (41.2%)	318 (44.5%)
I would like more institutional support or training on responsible LLM use	33 (4.6%)	37 (5.2%)	96 (13.4%)	250 (35.0%)	298 (41.7%)
I have ethical concerns about the use of LLMs in research in general	15 (2.1%)	39 (5.5%)	101 (14.1%)	298 (41.7%)	261 (36.6%)
LLM use may compromise the scientific integrity or rigour of research outputs	17 (2.4%)	70 (9.8%)	125 (17.5%)	296 (41.5%)	206 (28.9%)
Researchers should be required to disclose LLM use in academic writing	15 (2.1%)	37 (5.2%)	92 (12.9%)	183 (25.6%)	387 (54.2%)
I have concerns that LLM use could affect how my academic work is evaluated (eg, by peer reviewers or funding bodies)	21 (2.9%)	76 (10.6%)	186 (26.1%)	264 (37.0%)	167 (23.4%)
I believe LLMs will reduce the need for certain types of research roles (eg, research assistants, copy editors, reviewers)?	74 (10.4%)	184 (25.8%)	149 (20.9%)	203 (28.4%)	104 (14.6%)

LLM, large language model.

## Discussion

While prior work on LLMs in mental health has focused almost exclusively on clinical applications,^8^ less is known about their potential to support mental health research. To address this gap, we surveyed a diverse global sample of mental health researchers (n=714) from 42 countries across all career stages and varying research foci to examine their practices and perspectives on using LLMs in academic research.

We found evidence of widespread adoption of LLMs among mental health researchers. Nearly 70% of researchers reported using LLMs—most commonly ChatGPT—to assist with their academic research, indicating rapid uptake within the field. Adopters reported using LLMs most commonly for proofreading (69%) or for language-based support with coding (49%). Very few (<15%) used LLMs for more complex research tasks such as formal data analysis/visualisation, interpreting output, and assisting with study design development. This suggests that researchers currently view LLMs primarily as support tools for more technical or routine-level tasks, rather than for deeper analytical or methodological work which typically requires careful human oversight. LLM use was more common among researchers with fewer years of experience. This suggests that early career researchers may be more open to experimenting with emerging technologies, possibly due to greater digital fluency (ie, a higher comfort level and proficiency in navigating digital tools, adapting to new technologies, and integrating them into daily workflows) or perceived benefits in managing time and research output.^18^ In contrast, experienced researchers expressed more scepticism, were less familiar with these tools, and perceived greater risks in integrating LLMs into established workflows.

While we observed differences in adoption based on career stage and experience, it is also likely that broader contextual factors, such as institutional policies, language availability, internet infrastructure and data privacy regulations, play an important role in shaping LLM adoption across global regions. For instance, researchers in countries where institutional guidelines restrict AI tool use, or where LLMs are not well-supported in local languages, may encounter more barriers to integration. These factors potentially contribute to disparities in LLM adoption and warrant further investigation in future cross-cultural research.

Trust, ethical and accuracy concerns emerged as common themes among researchers. Over 40% of non-users cited these concerns as major factors in their decision not to use LLMs, while between 45% and 78% of users reported encountering similar issues during periods of use. These concerns are not unique to the mental health field, with prior survey studies revealing similar apprehensions among researchers across different career stages and disciplines.^16 19^ These concerns are understandable from the researcher’s viewpoint for several reasons. First, LLMs can produce ‘hallucinations’, where the model generates plausible but non-factual content. For instance, a prior study demonstrated high hallucination rates when LLMs were used to generate references for human-performed systematic reviews, with hallucination rates reaching 39% for ChatGPT models and 91% for Gemini.^20^ These findings also raise concerns about whether less experienced researchers, who appear to use LLMs more frequently, are adequately equipped to detect and correct errors generated by these tools. Second, because most researchers rely on third-party LLM platforms (eg, Open AI, Google Gemini), there is a legitimate risk of data leakage. These platforms often transmit and process data on external servers, raising concerns about how sensitive or proprietary research information is stored, accessed or used to train future models. These risks are particularly salient in mental health research that deals with sensitive health data, potentially intensifying concerns expressed by academics in this field. Third, LLMs are prone to biased outputs due to the nature of their training data, which often reflects societal inequalities and fails to uniformly represent diverse demographic groups.^21^ Consequently, LLMs may disproportionately reflect perspectives of more frequently occurring or dominant groups, posing risks to equity, representation and fairness in research outputs.

Beyond individual risks such as hallucinations or data leakage, broader ethical concerns also relate to transparency in authorship, unequal access to LLM tools and the risk of amplifying existing biases, particularly those affecting marginalised or under-represented populations. These issues are especially salient in mental health research, where LLMs trained on non-representative data may misinterpret or misrepresent the experiences of culturally diverse or structurally disadvantaged groups.^22^ Furthermore, inequitable access to advanced LLM platforms, due to cost, infrastructure or policy barriers, may widen global research disparities, limiting who can fully benefit from these tools.^23^ Therefore, responsible LLM integration requires technical safeguards and attention to equity and transparency in authorship attribution and research dissemination.

Another prominent theme identified across respondents was the strong desire for further training and resources to support using LLMs in mental health research. Nearly, 65% of non-users reported that access to such training would increase their likelihood of adopting LLMs, while 75% of the total sample agreed that more institutional support and guidance are needed to ensure appropriate and responsible use of these tools. Given the complexity of LLM technologies and the widespread concerns regarding bias, ethics and accuracy, there is a clear need for dedicated efforts to develop field-specific guidelines and training resources that promote safe, ethical and responsible use of LLMs for research purposes. This need is particularly urgent in light of our finding that nearly nine in 10 respondents expect LLMs to become standard academic tools within the next 5–10 years. While mental health researchers are not expected to become experts in LLM technologies, institutions may benefit from offering standardised training modules, preconference workshops or educational webinars that educate staff on their capabilities, ethical and privacy risks, and best practices for integrating these tools into academic workflows.

Expectations around LLM disclosure in academic research are still evolving, but clear guidelines are needed. Most respondents (~80%) agreed that researchers should be required to disclose LLM use in academic writing, yet only half of respondents reported feeling comfortable disclosing LLM use in their work. Furthermore, almost two-thirds expressed concern that LLM use could impact how their work is evaluated. These findings highlight the need for field-wide consensus on disclosure practices for both manuscript preparation and peer review. While leading journals such as those in the *Nature* series, the *Journal of the American Medical Association*,and the *New England Journal of Medicine* have begun encouraging AI disclosure,^24^ similar guidance is needed within mental health-specific journals to promote transparency and maintain trust in scholarly communication.

This study is not without its limitations. One limitation is the potential for self-selection bias, as researchers with a greater interest in or curiosity about AI tools may have been more likely to respond to the survey (hence reflecting the 19% response rate). Thus, the sample may not fully represent the broader population of mental health researchers, potentially overestimating rates of LLM awareness, use or acceptance. Efforts to broaden recruitment that capture more diverse researchers in future are needed.

A second limitation is that most respondents were based in high-income countries, most commonly Australia (24%), the USA (26%) and the UK (10%). Therefore, these findings may not be generalisable to researchers in low- and middle-income countries (LMICs), where differences in infrastructure, internet accessibility, research funding and data privacy norms may substantially influence LLM adoption, usage patterns and ethical concerns. These contextual factors could shape both practical engagement with LLMs and broader attitudes towards their integration in research workflows. Future research should specifically explore practices and perspectives in LMIC contexts to gain a more comprehensive and globally inclusive understanding of LLM use in mental health research.

A third limitation is that the survey was not formally piloted or subjected to cognitive testing. While items were informed by previous LLM-related surveys in other scientific disciplines^16^ and shaped by conceptual literature on LLMs in academic research,^14^ the absence of formal validation may limit the interpretability of the attitudinal items. Future studies would benefit from piloting and psychometric assessment to strengthen the reliability and validity of measures assessing perspectives on LLM use.

A fourth limitation concerns the design and structure of the survey items, which may have restricted the depth and nuance of participants’ responses. While we included free-text options alongside most closed-ended questions, the primary reliance on predefined response categories may have limited the ability to capture the full range of attitudes, concerns and use cases associated with LLMs. For example, we did not ask participants to rank the severity of their concerns (among non-users) or the frequency of specific use cases (among users), which may have offered more granular insight into perceived risks and practical integration. Similarly, attitudinal questions were framed as binary or Likert-type items, rather than asking participants to prioritise or rank their responses, which could have helped identify which issues matter most in shaping adoption and resistance. In addition, the initial survey framing defined LLMs using common examples (eg, summarising, responding to questions, generating ideas), which may have constrained participants’ thinking around more advanced or emerging capabilities. Thus, we may have missed opportunities to assess researchers’ awareness of novel directions in LLM development, such as ‘AI scientist’ models capable of automating entire research pipelines. Future studies should consider incorporating ranking tasks, open-ended exploration of speculative use cases and mixed methods designs to gain a more comprehensive understanding of how LLMs are being used and imagined within academic research.

### Clinical implications

In conclusion, this study offers timely insights into current practices, benefits and concerns surrounding LLM use in mental health research. While the adoption of LLMs in research is widespread, challenges and concerns related to trust, ethics, model accuracy and training are pervasive. Given the rapid pace of AI and LLM developments, adoption in this context will only increase. While the use of LLMs for routine research tasks should not be discouraged—particularly when they enhance efficiency and productivity—it remains essential that human researchers critically and carefully evaluate model outputs for accuracy and reliability. As AI tools continue to evolve, developing field-specific guidelines, offering standardised training resources, improving institutional support and establishing consensus around disclosure practices will ensure the responsible and equitable integration of LLMs into mental health research.

## Supplementary material

10.1136/bmjment-2025-301787 online supplemental file 1

## Data Availability

Data are available on reasonable request.
