# Supplementary material for: Role of large language models in mental health research: an international survey of researchers’ practices and perspectives
Source: BMJ Ment Health. 2025 Jun 12;28(1):e301787. doi: 10.1136/bmjment-2025-301787 (PMC12164621; doi:10.1136/bmjment-2025-301787)
Supplement: online supplemental file 1 [file bmjment-28-1-s001.docx]

**Supplementary Figure 1: Survey**

What is your age in years?

What gender do you identify with?

- Male
- Female
- Non-binary
- Other

What country do you currently live in?

What is your highest academic qualification?

- Bachelor's degree
- Master's degree
- PhD/Doctorate degree
- MD
- Other (please specify)

What is your primary field of expertise (select the one that best applies)?

- Psychology
- Psychiatry
- Neuroscience
- Computer science
- Public health
- Nursing
- Counselling
- Behavioral science
- Other (please specify)

What is currently your primary affiliation (select the one that best applies)?

- University/academic institution
- Hospital/healthcare setting
- Private sector
- Government agency
- Independent researcher
- Other (please specify)

Select the current job title/role that best describes you?

- PhD/doctoral student
- Post-doctoral researcher
- Lecturer/Assistant professor
- Associate Professor
- Professor
- Researcher-Scientist
- Clinician-scientist
- Other (please specify)

Approximately how many years of research experience do you have?

- Less than 1 year
- 1-5 years
- 6-10 years
- 11-15 years
- 16-20 years
- More than 20 years

What is your Google Scholar h-index (if known)?

- 0-10
- 11-20
- 21-30
- 31-40
- 41-50
- 51-60
- 61-70
- 71-80
- 81-90
- >91
- Prefer not to say
- I am not sure

Approximately how many peer-reviewed publications have you authored or co-authored?

- 0
- 1-20
- 21-40
- 41-60
- 61-80
- 81-100
- 100-200
- >200
- Prefer not to say
- I am not sure

What are your primary research methods? (Select all that apply)

- Quantitative (e.g., RCTs, surveys, observational studies)
- Qualitative (e.g., interviews, thematic analysis)
- Mixed methods (combination of quantitative and qualitative)
- Computational modeling or simulation
- Systematic reviews/meta-analysis
- Other (please specify)

What is your experience with artificial intelligence or machine learning in research?

- No experience
- Limited experience
- Some experience
- Extensive experience

What are your primary research areas within mental health sciences? (you can select all that apply)

- Depressive disorders
- Anxiety disorders
- Eating disorders
- Psychosis and/or schizophrenia
- Bipolar disorders
- Personality disorders
- Substance use disorders
- Neurodevelopmental disorders
- Trauma and PTSD
- Sleep disorders
- Other (please specify)

What is your primary research focus (you can select all that apply)?

- Treatment/intervention (e.g., psychotherapy, pharmacotherapy, digital interventions)
- Prevention and early intervention (e.g., risk reduction, resilience programs)
- Assessment & diagnosis (e.g., screening tools, clinical interviews, biomarker research)
- Epidemiology (e.g., prevalence, risk factors, population-based studies)
- Mechanisms of mental illness (e.g., neurobiological, psychological, genetic, or social determinants)
- Digital mental health & technology (e.g., mobile apps, telehealth)
- Health services and implementation research (e.g., access to care, service delivery, implementation science)
- Mental health policy & advocacy (e.g., legislative frameworks, policy evaluation, advocacy)
- Other (please specify)

“The following questions ask about your experience with and perspectives towards large language models (LLMs). LLMs are advanced artificial intelligence systems that can understand and generate human-like text. They are trained on vast amounts of written information and can respond to questions, summarize content, assist with writing, and generate ideas in a conversational way. Examples include ChatGPT, Google Gemini, and Claude.”

“Have you ever used a Large Language Model (LLM) to assist with any aspect of your academic research?”

No

Yes

“If answered NO, what are your main reasons for not using LLMs in research? (Select all that apply)”

- Lack of awareness
- No need for me to use AI tools like these
- I have trust concerns (such as its accuracy, biases etc.)
- I have ethical concerns about its use (such as plagiarism, responsible AI use, data privacy concerns)
- There are institutional restrictions I encounter
- I have preference for traditional non-AI methods for doing research
- I lack the technical skills to use these
- I lack access to those AI tools I would want to use
- I have concerns about the reliability or reproducibility of these tools
- Other (please specify)

Would you be more likely to use LLMs for your research if the following were addressed? (yes, no or unsure)

- Clear ethical guidelines for AI and LLM use
- Improved accuracy and reliability of LLMs
- Increased institutional support or endorsement for LLMs
- Better integration with existing research tools (e.g., citation managers, coding platforms) or workflows
- Greater transparency (e.g., clarity on sources, citations, and methods)
- Improved data privacy and security assurances
- More training or resources on how to use AI and LLMs effectively
- Clearer acceptance of AI and LLMs from journals and funding bodies

IF ANSWERED YES TO LLM US: “Which LLM(s) have you used for academic research? (Select all that apply)”

- ChatGPT (by OpenAI)
- Google Gemini (formerly Bard)
- Claude (by Anthropic)
- Meta AI (Llama series)
- Microsoft Copilot (formerly Bing Chat)
- Grok (X/Twitter)
- Perplexity AI
- Other (please specify)

“How frequently do you use LLMs for research-related tasks?”

- Daily
- Weekly
- Monthly
- Occasionally (less than once a month)
- Only used once or twice

“: For what purposes have you used LLMs in your research using the available options (Select all that apply)”

- Generating ideas for research questions or hypotheses
- Drafting or structuring research papers, abstracts, or grants
- Proofreading or improving writing clarity
- Assisting with peer-review of papers or grant applications
- Creating education materials or presentations
- Summarizing, organizing or synthesizing scientific literature
- Assisting with data cleaning or preparation
- Generating or refining code for statistical or computational analysis (e.g., R, Python, MATLAB)
- Analyzing qualitative data (e.g., thematic analysis, summarizing interview transcripts)
- Creating or refining data visualizations
- Assisting with study design or methodology development
- Suggesting relevant measures, scales, or assessments
- Interpreting or contextualizing statistical findings or research outcomes
- Interpreting other academic research papers
- Automating research-related tasks (e.g., formatting references, preparing tables)
- Supporting participant recruitment (e.g., drafting recruitment messages, generating survey questions)
- Summarizing meeting notes or research discussions
- Assisting with social media or science communication (e.g., drafting posts, plain-language summaries)
- None of these (please specify other purposes that do not fit into those options above)

“What challenges or limitations have you encountered when using LLMs for your research? (Select all that apply)”

- Inaccurate or misleading responses
- Lack of transparency about sources and citations
- Ethical concerns (e.g., data privacy/confidentiality, authorship, plagiarism)
- Bias in responses
- Over-reliance on AI-generated content
- Institutional or journal policies restricting LLM use
- Technical limitations (e.g., token/context length, inability to handle large datasets)
- I have not encountered any challenges/limitations

“Has using LLMs made your research process more efficient?”

- Yes
- Unsure
- No

“Has LLM use improved the quality of your research outputs?”

- Yes
- Unsure
- No

Would you recommend LLMs to colleagues for research support?

- Yes
- Unsure
- No

“Do you feel comfortable disclosing LLM use in academic work (e.g., acknowledgments, methods sections)?”

- Yes
- Unsure
- No

“FOR ALL PARTICIPANTS: Please indicate your level of agreement towards the following questions about LLM use in research (STRONGLY DISAGREE, SOMEWHAT DISAGREE, NEITHER AGREE NOR DISAGREE, SOMEWHAT AGREE, STRONGLY AGREE).”

- LLMs will become a standard tool in academic research within the next 5-10 years
- I would like more institutional support or training on responsible LLM use
- I have ethical concerns about the use of LLMs in research in general
- LLM use may compromise the scientific integrity or rigor of research outputs
- Researchers should be required to disclose LLM use in academic writing
- I have concerns that LLM use could affect how my academic work is evaluated (e.g. by peer reviewers or funding bodies)
- I believe LLMs will reduce the need for certain types of research roles (e.g., research assistants, copy editors, reviewers)?

| Supplementary Table S1  Characteristics of the total sample (N=714) | | | | | | | |
| --- | --- | --- | --- | --- | --- | --- | --- |
| **Variable** | **Data** |  | **Variable** | **Data** |  | **Variable** | **Data** |
| **Age** | 40.2 (11.48) |  | **Publications** |  |  | Belgium | 9 (1.3%) |
| **Gender** |  |  | 0 | 15 (2.1%) |  | New Zealand | 8 (1.1%) |
| Man | 277 (38.8%) |  | 1-20 | 233 (32.6%) |  | Japan | 7 (1.0%) |
| Women | 422 (59.1%) |  | 21-40 | 136 (19.0%) |  | France | 5 (0.7%) |
| Non-binary | 10 (1.4%) |  | 41-60 | 93 (13.0%) |  | Denmark | 5 (0.7%) |
| Other | 1 (0.1%) |  | 61-80 | 50 (7.0%) |  | India | 5 (0.7%) |
| Not specified | 4 (0.6%) |  | 81-100 | 43 (6.0%) |  | Israel | 4 (0.6%) |
| **Qualification** |  |  | 100-200 | 85 (11.9%) |  | Portugal | 4 (0.6%) |
| Bachelor’s degree | 39 (5.5%) |  | > 200 | 52 (7.3%) |  | Finland | 3 (0.4%) |
| Master’s degree | 86 (12.0%) |  | Prefer not to say/unsure | 7 (1.0%) |  | Czech Republic | 3 (0.4%) |
| PhD/Doctorate | 543 (76.1%) |  | **Primary Research Focus** |  |  | Austria | 3 (0.4%) |
| MD | 27 (3.8%) |  | Quantitative | 602 (84.3%) |  | Brazil | 3 (0.4%) |
| Other | 19 (2.7%) |  | Qualitative | 194 (27.2%) |  | Hungary | 2 (0.3%) |
| **Field of Expertise** |  |  | Mixed methods | 253 (35.4%) |  | Ireland | 2 (0.3%) |
| Psychology | 503 (70.4%) |  | Computation modelling | 57 (8.0%) |  | Nigeria | 2 (0.3%) |
| Psychiatry | 88 (12.3%) |  | Review/meta-analysis | 266 (37.3%) |  | Romania | 2 (0.3%) |
| Neuroscience | 21 (2.9%) |  | Other | 18 (2.5%) |  | Slovenia | 2 (0.3%) |
| Computer science | 10 (1.4%) |  | **Field of Study** |  |  | Turkey | 2 (0.3%) |
| Public health | 31 (4.3%) |  | Depression | 325 (45.5%) |  | Argentina | 1 (0.1%) |
| Nursing | 2 (0.3%) |  | Anxiety/related disorders | 356 (49.9%) |  | Cyprus | 1 (0.1%) |
| Counselling | 10 (1.4%) |  | Psychosis/schizophrenia | 100 (14.0%) |  | Ethiopia | 1 (0.1%) |
| Behavioral science | 12 (1.7%) |  | Eating disorders | 164 (23.0%) |  | Indonesia | 1 (0.1%) |
| Other | 37 (5.2%) |  | Bipolar disorders | 67 (9.4) |  | Korea (South) | 1 (0.1%) |
| **Primary Affiliation** |  |  | Personality disorders | 50 (7.0%) |  | Lebanon | 1 (0.1%) |
| University | 615 (86.1%) |  | SUDs/addiction | 90 (12.6%) |  | Malaysia | 1 (0.1%) |
| Hospital/healthcare setting | 63 (8.8%) |  | Neurodev disorders | 73 (10.2%) |  | Qatar | 1 (0.1%) |
| Private sector | 16 (2.2%) |  | Trauma/PTSD | 134 (18.8%) |  | Singapore | 1 (0.1%) |
| Government agency | 9 (1.3%) |  | Sleep disorders | 36 (5.0%) |  | South Africa | 2 (0.3%) |
| Independent researcher | 5 (0.7%) |  | Suicide/self-harm | 30 (4.2%) |  | Taiwan | 1 (0.1%) |
| Other | 6 (0.8%) |  | Other | 130 (18.2%) |  | Uganda | 1 (0.1%) |
| **Job Title** |  |  | **Research Emphasis** |  |  | Did not say | 6 (0.8%) |
| PhD/Doctoral/MA student | 137 (19.2%) |  | Treatment/intervention | 433 (60.6%) |  |  |  |
| Post-Doc/Research Fellow | 101 (14.1%) |  | Prevention/risk factor | 258 (31.6%) |  |  |  |
| (Senior)Lecturer/Assist Prof | 113 (15.8%) |  | Assessment/diagnosis | 241 (33.8%) |  |  |  |
| Associate Professor | 129 (18.1%) |  | Epidemiology | 177 (24.8%) |  |  |  |
| Professor | 114 (16.0%) |  | Mechanistic research | 347 (48.6%) |  |  |  |
| Researcher-Scientist | 50 (7.0%) |  | Technology & digital health | 226 (31.7%) |  |  |  |
| Clinician-scientist | 54 (7.6%) |  | Services/implementation | 192 (26.9%) |  |  |  |
| Other | 16 (2.2%) |  | Policy & advocacy | 71 (9.9%) |  |  |  |
| **Years Research Experience** |  |  | Other | 21 (2.9%) |  |  |  |
| < 1 year | 3 (0.4%) |  | **AI/ML experience** |  |  |  |  |
| 1-5 years | 151 (21.1%) |  | No experience | 132 (18.5%) |  |  |  |
| 6-10 years | 185 (25.9%) |  | Limited experience | 329 (46.1%) |  |  |  |
| 11-15 years | 160 (22.4%) |  | Some experience | 216 (30.3%) |  |  |  |
| 16-20 years | 83 (11.6%) |  | Extensive experience | 37 (5.2%) |  |  |  |
| > 20 years | 132 (18.5%) |  | **Country of Residence** |  |  |  |  |
| **Google Scholar h-index** |  |  | United States | 186 (26.1%) |  |  |  |
| 0-10 | 179 (25.1%) |  | Australia | 171 (23.9%) |  |  |  |
| 11-20 | 130 (18.2%) |  | United Kingdom | 73 (10.2%) |  |  |  |
| 21-30 | 107 (15.0%) |  | Canada | 43 (6.0%) |  |  |  |
| 31-40 | 60 (8.4%) |  | Germany | 32 (4.5%) |  |  |  |
| 41-50 | 37 (5.2%) |  | Netherlands | 31 (4.3%) |  |  |  |
| 51-60 | 24 (3.4%) |  | Sweden | 18 (2.5%) |  |  |  |
| 61-70 | 12 (1.7%) |  | Spain | 15 (2.1%) |  |  |  |
| 71-80 | 9 (1.3%) |  | Norway | 15 (2.1%) |  |  |  |
| 81-90 | 10 (1.4%) |  | Switzerland | 14 (2.0%) |  |  |  |
| > 91 | 12 (1.7%) |  | Italy | 13 (1.8%) |  |  |  |
| Prefer not to say/unsure | 134 (18.8%) |  | China | 13 (1.8%) |  |  |  |


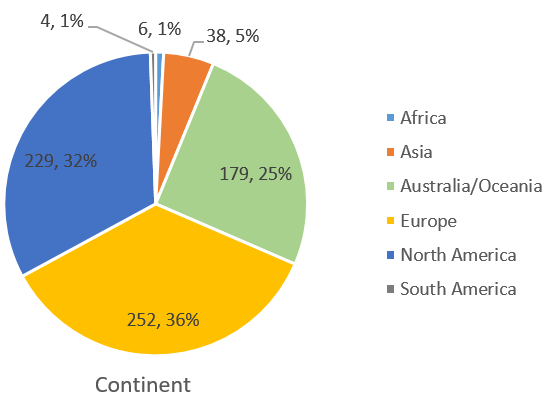

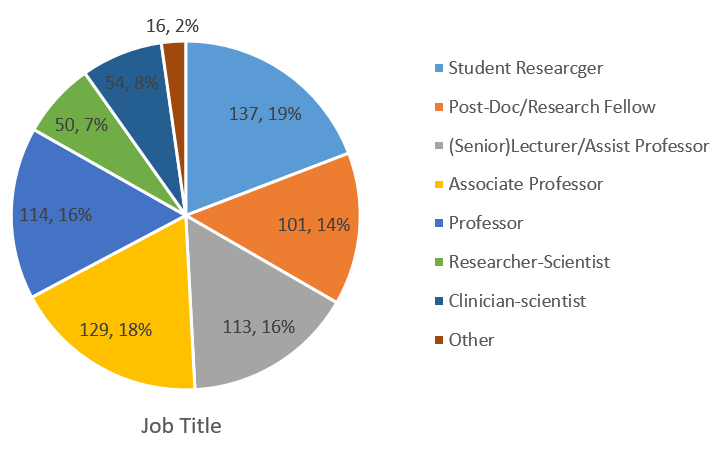


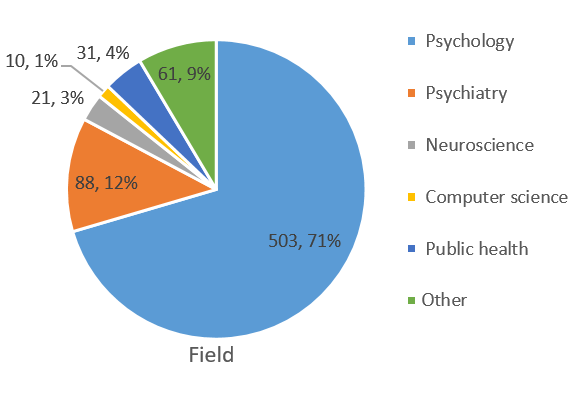


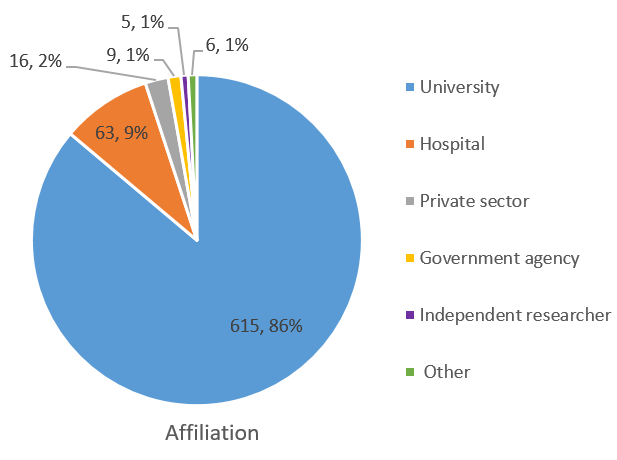


Supplementary Figure 2: Breakdown of Participant Characteristics (N=714)


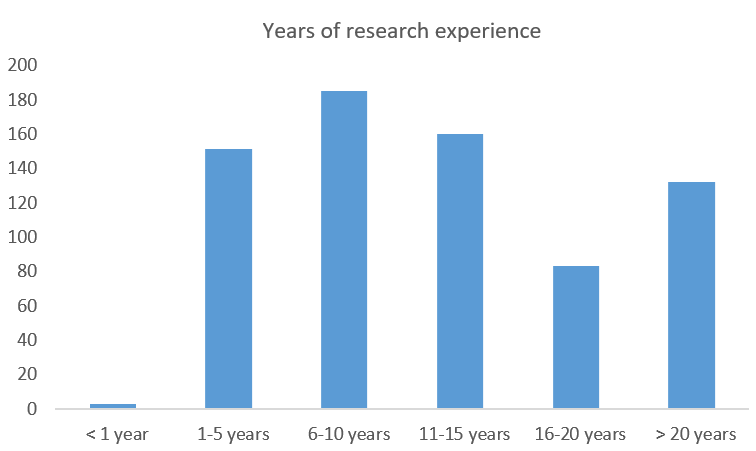

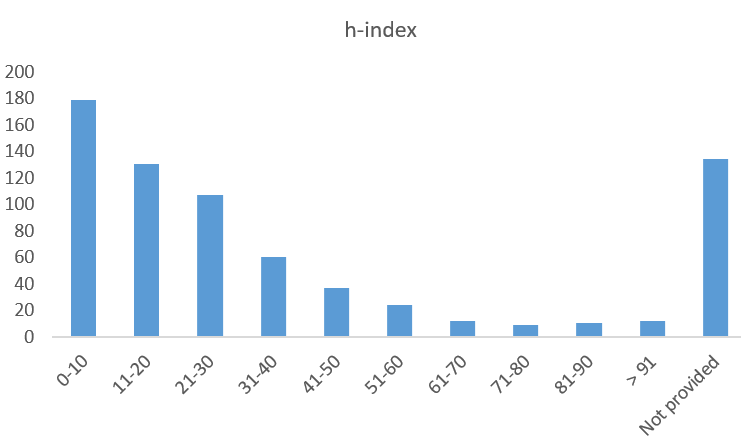


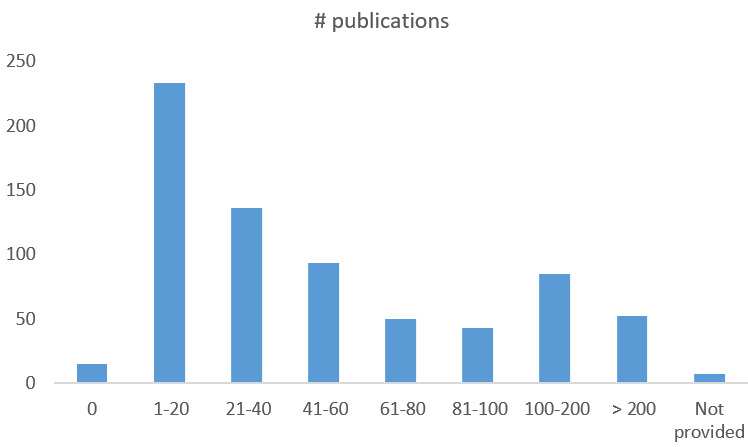


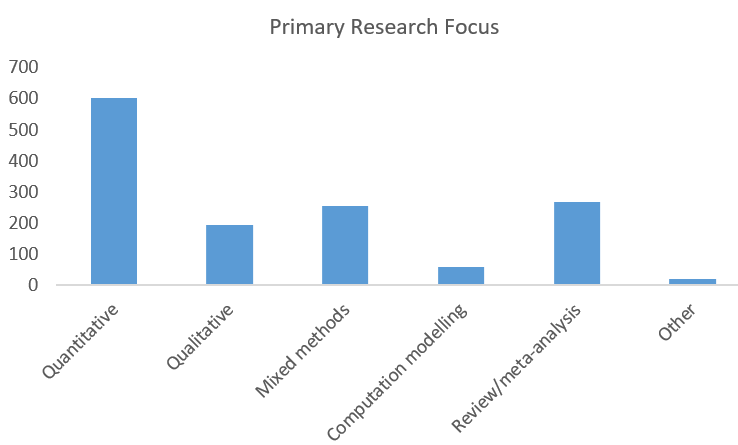


Supplementary Figure 3: Breakdown of Participant Research Experience Characteristics

Note that participants could select more than 1 primary research focus.


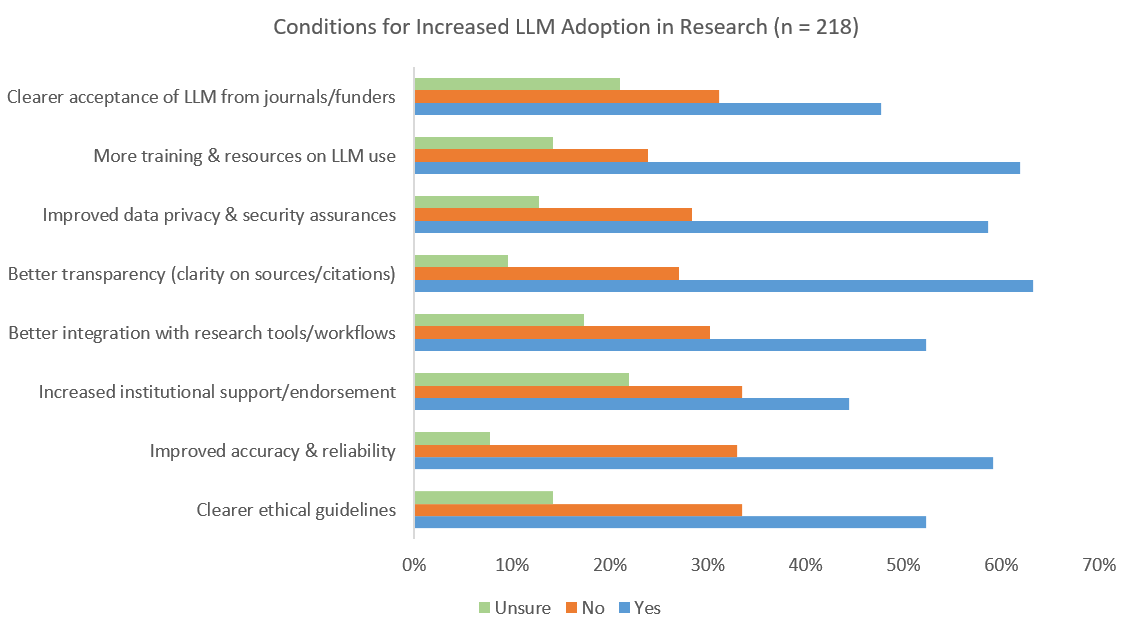


Supplementary Figure 4: Conditions for Increased Adoption of LLM in Research among Non-Users (n = 218).
